# Cross-sectional associations between vitamin D status and periodontitis in adults in Northern Norway: The Tromsø Study 2015–2016

**DOI:** 10.1186/s12903-025-06261-2

**Published:** 2025-05-30

**Authors:** Fanny Maria Lindholm, Birgitta Jönsson, Magritt Brustad

**Affiliations:** 1 Department of Community Medicine, UIT The Arctic University of Norway, Tromsø, Norway; 2 The Public Dental Health Service Competence Centre of Northern Norway, Tromsø, Norway; 3 Department of Periodontology, Institute of Odontology, The Sahlgrenska Academy, University of Gothenburg, Gothenburg, Sweden

**Keywords:** Epidemiology, Inflammation, Observational Study, Periodontal Diseases, Periodontitis, 25-hydroxyvitamin D

## Abstract

**Background:**

Vitamin D is involved in the immune system by regulating inflammatory processes. Over the recent decades, there has been an increase in studies investigating the potential influence of vitamin D status on inflammatory diseases and conditions. However, research on the link between vitamin D and periodontitis remains scarce. Hence, we aimed to examine the relationship between serum 25(OH)D levels and periodontitis in an adult population in Northern Norway.

**Methods:**

The study was based on cross-sectional data from a population-based study: the seventh Tromsø study, conducted in 2015 – 2016. Eligible participants were adults ( ≥ 40 years old) who had both a valid periodontal diagnosis and data on serum 25(OH)D levels, giving a total study sample of *n* = 3,693 participants (51.4% women). Periodontitis was defined according to the 2017 AAP/EFP classification system. Two groups were compared: no-periodontitis/stage I periodontitis versus stages II-III/IV periodontitis. Using a bivariate logistic regression analysis, the association between these two groups and serum 25(OH)D was tested, controlling for age, sex, smoking, toothbrushing frequency, and socioeconomic status. Periodontal pockets of ≥ 5 mm and their association with serum 25(OH)D were also tested.

**Results:**

About 89% were classified as having periodontitis, and 51.6% had stages II–III/IV periodontitis. About 4% of the participants were at deficient levels of serum 25(OH)D (< 30 nmol/L). Levels of serum 25(OH)D of < 30 nmol/L were associated with periodontitis stage II-III/IV (odds ratio [OR] 1.53, 95% confidence interval [CI]: 1.03–2.28). Stratified by when the season serum 25(OH)D was measured (March-September vs October-February), the summer strata was significantly associated with periodontitis II-III/IV (30–50 nmol/L = OR 1.31, 95% CI:1.05–1.63, *p* = 0.018). Vitamin D deficiency and insufficiency were associated with having at least one periodontal pocket of ≥ 5 mm (< 30 nmol/L = 1.78, 95% CI:1.22–2.60, *p* = 0.003: 30–50 nmol/L = OR was 1.23, 95% CI:1.03–1.47, *p* = 0.020, respectively).

**Conclusion:**

In this Norwegian adult population, blood 25-hydroxy vitamin D [25(OH)D] levels were associated to periodontitis stage II-III/IV and a periodontal pocket depth of ≥ 5 mm.

## Introduction

Periodontitis is a highly prevalent chronic inflammatory disease that affects the supporting tissues [1] of the teeth in response to bacterial colonisation on the tooth surface. Advanced periodontitis has been estimated to affect approximately 10% of the global population. In Norway, about 50% of the population has periodontitis, and 9–20% have an advanced form of periodontitis, depending on which case definition has been used [1–3]. Clinical signs of periodontitis are swollen, reddened, and bleeding gingiva, but is distinctly characterised by the destruction of the supporting tissues around the affected teeth (the gingiva, bone and periodontal ligament), which may ultimately lead to tooth loss [4, 5]. Periodontitis is initiated and sustained by dysbiosis of the commensal oral microbiota in the dental plaque biofilm. Alternatively, it may also be due to an excessive response from the host to the presence of microbes. This subsequently results in a heightened inflammatory state, which in turn causes tissue damage and disease manifestation [5].

Periodontitis contributes to systemic inflammation by promoting bacteraemia during personal oral hygiene, chewing or dental treatment [6, 7]. In more severe periodontitis, there has been a noted increase in the concentrations of pro-inflammatory cytokines, coupled with a decrease in anti-inflammatory cytokines [4]. This imbalance between inflammatory and anti-inflammatory processes could account for the excessive inflammatory response observed in periodontitis. Furthermore, it could elucidate why an acute inflammatory reaction evolves into chronic inflammation. In addition, specific cytokines, such as IL-1β and TNF-α, have been found to activate osteoclasts, a particular type of bone cell responsible for the breakdown and remodelling of old and damaged bone tissue [4, 5].

Risk factors for periodontitis are tobacco smoking, psychological stress and depression, alcohol intake, obesity, diabetes, metabolic syndrome, hormones, and osteoporosis [8–12]. Adhering to several healthy lifestyle practices simultaneously, like not smoking, exercising, keeping a healthy BMI, and eating a nutritious diet, have been shown to lower the risk of developing periodontitis and losing teeth [8]. Consequently, it is crucial to identify potential factors that promote periodontal health and factors that reduce the risk of periodontitis.

Vitamin D is an essential micronutrient primarily known for its important role in calcium absorption and homeostasis for proper bone mineral matrix formation and skeletal health [13]. As the enzyme for activating vitamin D into its active metabolite and vitamin D receptors have been identified in several tissues and organs in the body, the role of vitamin D most likely goes beyond skeletal health and calcium metabolism [14].

The recommended intake of vitamin D for adults in the Nordic countries has been set to 10 µg/day [15]. The metabolite 25-hydroxy vitamin D [25(OH)D] is the well-established biomarker for vitamin D status measured in blood [13]. Generally, people are considered vitamin D deficient at serum 25(OH)D concentrations less than 30 nmol/L and potentially at risk of vitamin D inadequacy at 30 to 50 nmol/L and a concentration of 25(OH)D at more than 50 nmol/L has been suggested as optimal [16]. The two primary forms of vitamin D are *cholecalciferol* (D_3_) and *ergocalciferol* (D_2_) [13]. For most populations vitamin D is primarily obtained from exposure to ultraviolet (UV) B radiation from sunlight (in the form of D_3_) [17], and additionally through nutritional supplements or the diet (as D_2_ and D_3_). However, only a few foods are good sources of vitamin D. In Norway, the primary sources of vitamin D include fatty fish or fish products rich in fish fats, along with fortified dairy products like e.g. margarine and butter [18]. In addition, some mushrooms, such as chanterelles, contain some vitamin D_2_.

In the last two decades, prevalence rates of vitamin D deficiency (25(OH)D < 30 nmol/L) in Europe has been estimated as 13%, and 40% for vitamin D insufficiency (25(OH)D < 50 nmol/L) [19]. Deficiency can be developed due to low dietary intake, limited sun exposure, or diseases or conditions that affect vitamin D metabolism or uptakes. Generally, groups with increased risk in the Nordic countries are non-Western immigrants, dark-skinned people, people who do not eat fatty fish or fortified food products, adolescents, and older adults, especially those getting limited sun-shine exposure [18].

Interestingly, data from Northern Norway have shown that vitamin D status is sufficient for most of the population [20]; however, there are still risk groups where 25(OH)D levels are insufficient. There is evidence for a general drop in 25(OH)D concentrations during winter months [21], confirming the contribution of sun-induced vitamin D for the population. Teenagers and young adults [22], non-Western immigrants and people who consume little or no foods containing vitamin D have been identified as vulnerable groups for vitamin D deficiency or insufficiency in Norway [18].

Biological evidence shows that vitamin D plays a role in regulating specific leukocytes and cell functions in both innate and adaptive immune responses. Vitamin D can influence and adjust the inflammatory process by regulating the dendritic cell function and the production of inflammatory cytokines and by inhibiting the reproduction of pro-inflammatory cells [23, 24]. Additionally, VDR expression and activity have been found to affect T-lymphocyte development, differentiation, and effector function, which play an essential role in the adaptive immune system [25].

Although vitamin D's role in immune-related health and diseases is not fully understood, and some findings are conflicting, several observational studies have reported an association between periodontitis and vitamin D levels [26–28]. This association may be linked to the role of vitamin D in the immune system. It is shown that vitamin D enhances the production of anti-microbial peptides, potentially reducing the exposure of periodontal tissues to oral bacteria. Additionally, vitamin D may suppress pro-inflammatory cytokines such as TNF-α and IL-6, which contribute to the pathway that inhibits the differentiation of osteoclast progenitor cells [29].

We aimed to examine the relationship between levels of serum 25(OH)D and periodontitis in adults in Northern Norwegian in a large population-based epidemiological study, testing the hypothesis that individuals with more advanced periodontitis in this population would have more vitamin D deficiency and insufficiency compared to those with mild or no periodontitis.

## Methods

### The Tromsø study

Analysis was based on cross-sectional data from an adult Norwegian population participating in the seventh survey of the Tromsø Study.

The Tromsø study is a population-based cohort study comprising seven waves of data collection. The first was completed in 1974, and the seventh and most recent (Tromsø 7) was conducted between March 2015 and November 2016 [30]. The Tromsø study consists of a comprehensive collection of health data from questionnaires and measurements, biological samples, and clinical examination.

In Tromsø 7, all inhabitants of Tromsø municipality from 40 years and above were invited to participate. Of the total 32 591 invited, 21 083 persons (64%) aged 40–99 years from urban and rural areas agreed to attend the survey. In total, 52,5% of the participants were female and 47,5% male [30]. The study population was representative of the general 2016 Tromsø population in the distribution of sex and age. A random subsample of those who participated also attended a dental examination (*n* = 3943) [30].

Eligible for our study were participants with data from both the biological samples and the dental examination. We excluded participants with missing values on periodontal status (*n* = 226) or missing data on vitamin D status (*n* = 24), given a total sample of *n* = 3693.

Tromsø7 was conducted in accordance with The Declaration of Helsinki and approved by the Regional Committee of Medical and Health Research Ethics North (reference 2014/940) and the Norwegian Data Protection Authority (reference 14/01463–4/CGN). All participants provided written informed consent.

### Dental examination and periodontitis case definition

Six calibrated dental hygienists performed the dental examination. The examination included an orthopantomogram (OPG), measuring of periodontal pocket depth (PPD) and bleeding on probing (BOP). The PPD was measured to the closest millimetre using a periodontal probe with single-millimetre graduations (WHO-probe LM555B) at four sites per tooth for all teeth and registration of BOP. Radiographic marginal bone loss (BL) was assessed on the orthopantomogram [30]. Bone loss of interproximal surfaces of all teeth, excluding third molars, was measured linearly with a transparent plastic ruler on the orthopantomogram as described by Holde et al. [31]. Periodontitis cases were defined according to the 2017 EFP/AAP periodontitis definition: stage I, stage II and stage III-IV periodontitis [32]. Two groups were created for the analysis: 1) periodontal healthy/gingivitis/stage I cases were merged into one group labelled “No-periodontitis/stage I” and 2) stage II and stage III/IV into one group labelled “periodontitis stage II-III/IV”. The PPD variable total number of periodontal pockets of ≥ 5 mm/individual was categorised into two new variables. One where the total number of PPD was divided into three groups: i) 0 PPD ≥ 5 mm; ii) 1–2 PPD ≥ 5 mm, and iii) ≥ 3 PPD ≥ 5 mm, and one with two categories: yes (at least one PPD ≥ 5 mm) and no (none).

### Vitamin D status

Levels of serum 25(OH)D (nmol/L) were measured in blood samples from the participants. All collected blood samples were analysed within 24 h in a laboratory at the University Hospital in Tromsø. The serum samples were processed after 30–60 min at room temperature [30]. Total serum 25(OH)D levels were measured using liquid chromatography-tandem mass-spectrometry (LCMS-MS), which measured both the separate and the collective sum of serum 25(OH)D_2_ and serum 25(OH)D_3_ in each blood sample [33]. The vitamin D metabolites [25(OH)D_2_ and 25(OH)D_3_] were separated and measured quantitatively in a liquid–liquid extraction using methanol and isopropanol. In the present study, the sum of 25(OH)D_2_ and 25(OH)D_3,_ hereafter referred to as serum 25(OH)D levels or vitamin D status, were divided into three groups—less than 30 nmol/L, 30–50 nmol/L, and greater than 50 nmol/L—in accordance with the suggested thresholds for deficiency, insufficiency, and sufficiency, respectively [16]. The date of attendance refers to the date when blood samples were taken. Attendance dates were split into two groups: sunnier months from March 1st to September 30th and less sunny months from October 1st to February 29th.

### Questionnaires

The data were collected using three questionnaires (Q1 and Q2) and one additional dietary frequency questionnaire, all included in the Tromsø 7 study. Questions covered socio-demographics, mental and somatic health, lifestyle, and well-being [30]. The variables from the questionnaire employed in the current study were smoking, toothbrushing, education, and household income.

One question assessed smoking status: “Do you/did you smoke daily?” (no never, yes now, or yes previously). One question assessed the frequency of toothbrushing: “How often do you brush your teeth?” with six response options: < 1 time/week, 1 time/week, 2–3 times/week, 4–6 times/week, 1 time/day, and 2 times/day or more often. These were merged into “once a day or less often” and “twice a day or more often” groups. Education was reported based on the highest completed education level, with response options: Primary/partly secondary education (Up to 10 years of schooling); Upper secondary education (a minimum of 3 years); College/university less than 4 years; and College/university 4 years or more. The two college/university level education groups in the education variable were merged into one category labelled “Tertiary” (education). The household income was assessed by one question: 'What was your household's total gross income in the last year?' with eight response options ranging from < 150,000 NOK to > 1,000,000 NOK. The household income variable was divided into four income groups with split points at 350 000 NOK, 551 000 NOK, and 751 000 NOK.

Age was grouped into 10-year age groups of 40–49, 50–59, 60–69, and 70 + . As there were few participants over 80 (3,1%), all participants aged 70 and up were merged.

### Statistical analysis

Descriptive statistics were tested using Pearson’s chi-square test. Three sets of binary multiple logistic regression analyses were conducted to examine the associations between levels of vitamin D status (deficiency, insufficiency, and optimal) and periodontitis. Odds ratio (OR), confidence intervals (CI 95%) and *P*-values were extracted from all regression analyses. Wald statistic was used for each variable to test and compare their importance for the model.

The first regression analysis used periodontitis as the dependent variable: No periodontitis/stage I vs Periodontitis stages II-III/IV. The independent variables were vitamin D status, sex, 10-year age groups, smoking status, toothbrushing frequency, education, and income. All independent variables were categorical. Reference categories were chosen from those presumed to be at the lowest risk of more severe periodontitis, in accordance with the study hypothesis, and previously stated risk factors [8–10], to ensure positive Odds Ratios (OR) and facilitate a more straightforward interpretation of the results. Missing data on smoking, toothbrushing, education, and income (*n* = 254) were excluded in the respective analyses. Assumptions for the regression analyses were checked by assessing correlations and collinearity for all variables included in the models.

In an additional regression analysis, the dataset was split according to the attendance seasons (March-September or October-February), and the regression analysis was performed separately for these two groups. This complementary analysis examined whether seasonal sun exposure might influence the association between measured vitamin D status and periodontitis status. The dependent and independent variables were otherwise introduced in the same way as in the former analysis. Cases with missing data for smoking, toothbrushing, education, or income (*n* = 80 out of 1195) in the winter strata and (*n* = 174 out of 2498) in the March-September strata were excluded from the analysis.

Lastly, in the third binary logistic regression analysis, the variable number of periodontal pockets of 5 mm or more was used as the dependent variable. The PPD variable was dichotomised into at least one periodontal pocket of 5 mm or more versus none. Vitamin D status, sex, age groups, and smoking were used as independent variables. Participants with missing data on smoking (*n* = 70) were excluded from the analysis.

All statistical analyses were done using the statistical program IBM SPSS software version 28. Statistical significance was considered at the 5% level.

## Results

The study sample (*n* = 3693) participants consisted of 1898 (51.4%) females and 1795 (48.6%) males. In total, 89.2% of the participants had periodontitis, of which 10.8% were classified as stage I, 31.8% as stage II, and 19.8% as stage III-IV. In Table 1, serum 25(OH)D level, sex, age, education level, socioeconomic status, smoking and toothbrushing are presented in relation to periodontal status. A higher proportion of individuals in the older age group had periodontitis stages II-III/IV compared to those in other age groups. The distribution of vitamin D status categories did not differ between the two periodontal status groups (*P* = 0,537). All other included characteristics differed statistically between the two groups.

**Table 1:**
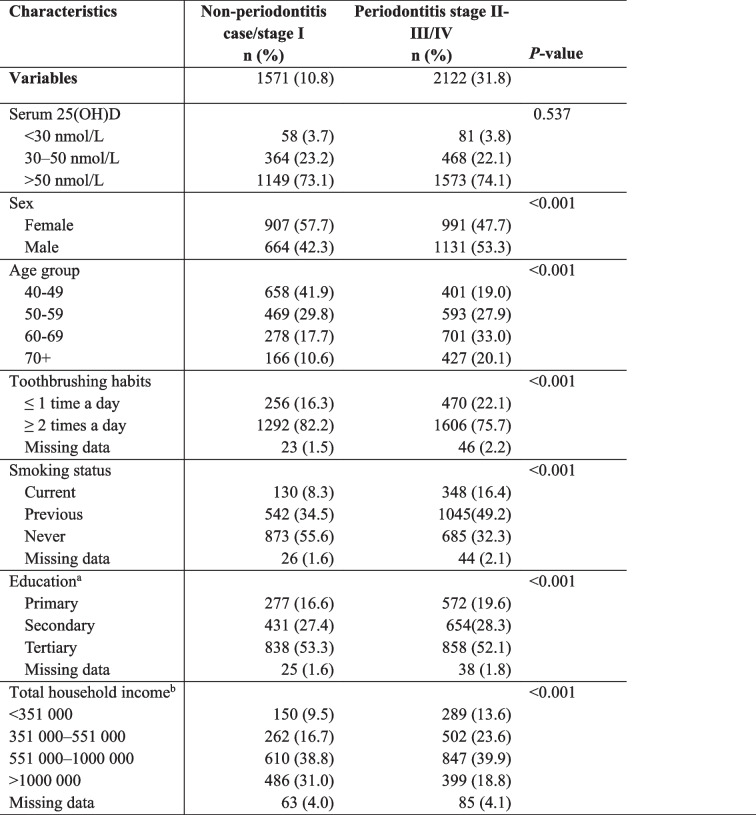
The distribution of Serum 25(OH)D, sociodemographic and behavioural factors in relation to periodontitis *n*=3693

Serum 25(OH)D are calculated as the sum of 25OHD2 and 25(OH)D3. P-values were calculated as Pearson’s Chi-square

^a^Primary: Up to 10 years of primary education, Secondary: 1-3 years of upper secondary education, Tertiary: University or college education

^b^In NOK

In Table 2, the distribution of serum vitamin D status following the suggested thresholds for deficiency (< 30 nmol/L), insufficiency (30–50 nmol/L) and sufficiency (> 50 nmol/L) are presented in relation to sociodemographic factors, education, income, behavioural factors, and number of PPD ≥ 5 mm. Most participants had sufficient serum 25(OH)D levels (73.7%), whereas 3.8% were classified as deficient. The majority (67.6%) of the blood samples were collected from March to September when the population was most exposed to sunlight.

**Table 2:**
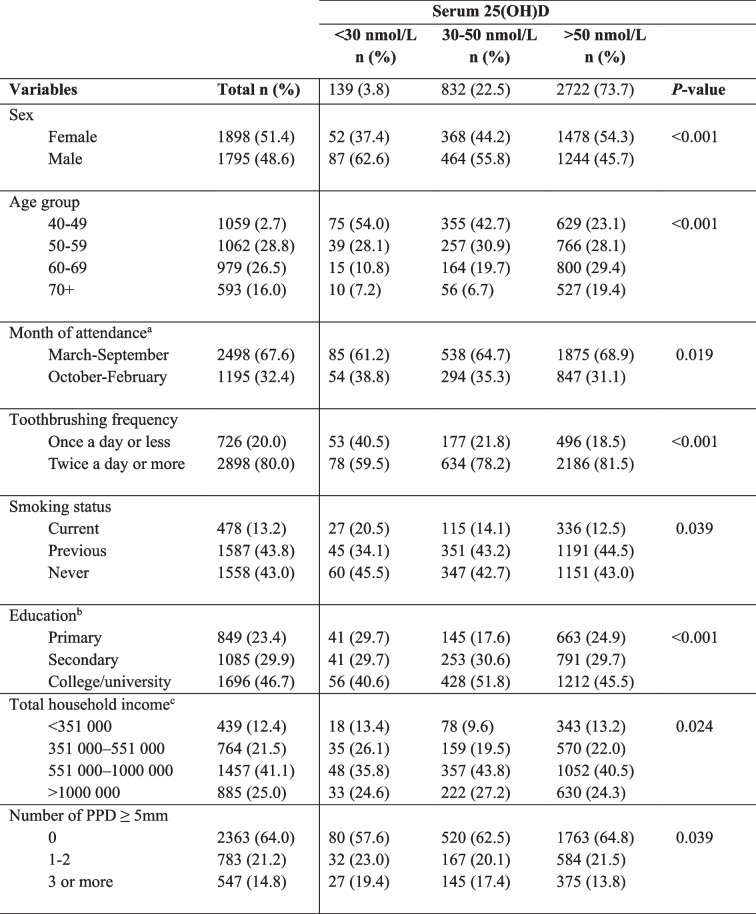
The distribution of serum vitamin D status following the suggested thresholds for deficiency, insufficiency and sufficiency in relation to sociodemographic factors, behaviours, and periodontal pocket depth (PPD)

Results are presented as frequencies and proportions within the respective serum 25(OH)D levels. P-values were calculated as Pearson’s Chi-square

^a^Derived from date of attendance for the first health examination

^b^Primary: Up to 10 years of primary education, Secondary: 1-3 years of upper secondary education, Tertiary: University or college education

^c^In NOK

### Regression model between periodontitis and Vitamin D status (serum 25(OH)D level)

The collinearity analysis gave a condition index of < 30 for all dimensions, although with a slight correlation between the two variables, education and income (values > 0.50) at the fifth dimension. However, the Pearson correlation coefficient for these two variables was 0,390, indicating that the correlation was acceptable (value < 0.7), and thus both were kept in the analyses. The variance inflation factor (< 10) and tolerance (> 0,1) indicated no collinearity and, hence, no objection to the regression analyses.

In the adjusted regression analysis (Table 3), periodontitis was significantly associated with vitamin D status (*P* = 0.036 and 0.038). Toothbrushing frequency and education were not statistically significant (*P* > 0.05), nor was the income category of < 351 000 NOK. The Wald-test values indicated that age was the most important predictor for severe periodontitis in this study sample, with the largest value (198.8), followed by smoking (Wald statistic = 135.5) and sex (Wald statistic = 47.9). For vitamin D status, the Wald statistic was 7.6.

**Table 3:**
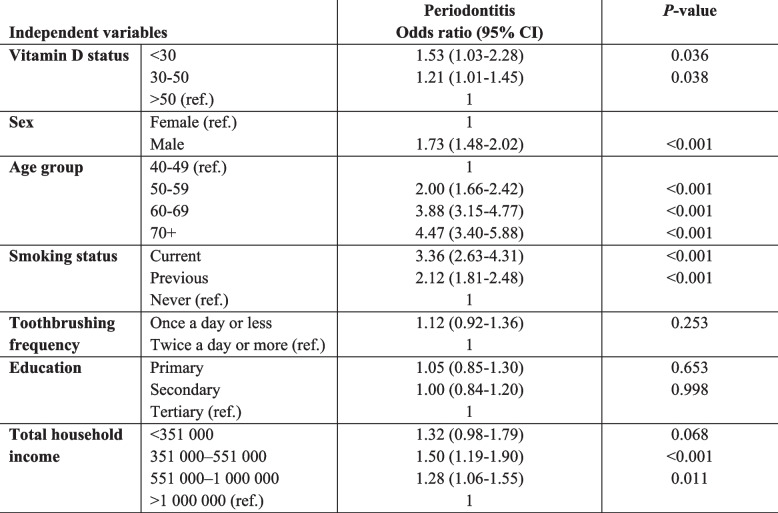
Odds^a^ for selected characteristics by periodontitis stages dichotomised into No-periodontitis/stage I vs Periodontitis stages II-III/IV^b^

^a^All variables were mutually adjusted for each other

^b^Subgroup totals may not add up to *n*=3963 due to missing values (see Table 2 for details). ^a^No periodontitis/stage I(n=1470) vs. Periodontitis stages II-IV (n=1969). Ref ref.: Reference category. Adjusted for 10-year age groups (ref.: 40-49 years), sex (ref.: female), smoking status (ref.: never), toothbrushing frequency (ref.: twice a day or more), education (ref.: tertiary), income (ref.:>1,000,000)

When conducting stratified analysis by season for the blood sampling (October-February and March-September), there was no statistical significance between periodontitis and vitamin D status when adjusting for sex, age, smoking, toothbrushing frequency, education, and income for the winter strata (data not shown). For the March-September strata, the OR for vitamin D status < 30 was 1.63 (95% CI:1.00–2.68, *P* = 0.052), and for vitamin D status 30–50, the OR was 1.31 (95% CI:1.05–1.63, *P* = 0.018).

### Regression model between having at least one PPD of ≥ 5 mm and Vitamin D status (serum 25(OH)D level)

Vitamin D deficiency and insufficiency were significantly associated with having at least one PPD ≥ 5 mm when adjusting for sex, age, and smoking. The odds of having serum vitamin D levels < 30 nmol/l compared to having serum vitamin D levels > 50 nmol/l was 1.78 (95% CI: 1.22–2.60, *P* = 0.003) for those with one or more PPD ≥ 5 mm (Table 4). In this analysis, smoking was the most important predictor (Wald statistic = 131.2). Wald’s statistic for vitamin D status was 12.6.

**Table 4:**
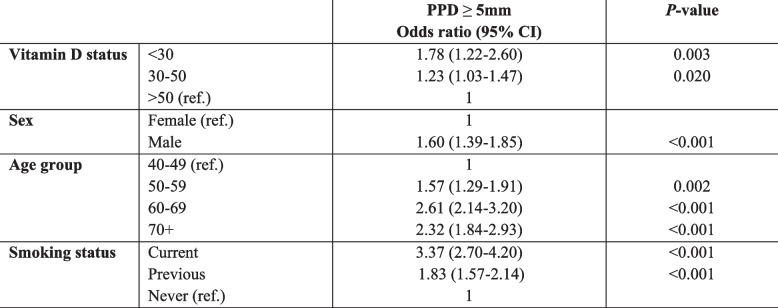
Odds of having at least one PPD ≥ 5mm versus having no PPD ≥ 5mm for selected characteristcs *n*=3623^a^

Adjusted for 10-year age groups (ref.: 40-49), sex (ref.: female) and smoking status (ref.: never)

*Ref. *Reference category

^a^No teeth with PPD ≥ 5 mm (*n* = 2322) vs. one or more PPD ≥ 5 mm (*n* = 1301)

## Discussion

We found that vitamin D status was associated with periodontitis stages II-III/IV. Persons with periodontitis stages II-III/IV had 53% higher odds of having deficient vitamin D levels than those in the non-periodontitis/stage i-group. Further, vitamin D deficiency and insufficiency were significantly associated with having PPD ≥ 5 mm. The month when blood samples had been collected was a significant predictor for serum 25(OH)D levels. When stratified by season, only the summer strata showed a significant association between vitamin D levels in the blood and periodontitis.

Our findings correspond with other published cross-sectional studies from extensive population-based surveys assessing periodontitis and serum vitamin D, like the Norwegian HUNT study on 25(OH)D levels [34] and the US-based NHANES study [35]—however, the meta-analysis conducted by Liang et al. [36] indicates that while observational evidence linking vitamin D levels to periodontitis is solid and consistent, the clinical evidence supporting vitamin D supplementation as an adjunct to periodontal therapy is relatively weak and inconsistent.

In the initial descriptive analyses, we observed an equal proportion of vitamin D deficiency between participants with no periodontitis or stage I periodontitis and those with stage II-III/IV periodontitis. However, after adjusting for confounding factors in the regression analysis, we found a statistically significant increase in the odds of vitamin D deficiency among those diagnosed with stage II to IV periodontitis. Therefore, differences in age distribution may explain the lack of difference in vitamin D deficiency proportions in the initial analysis. Vitamin D deficiency was most prevalent in the 40–49 age group; this group also comprised most cases of no-periodontitis/stage I periodontitis. The most likely explanation for this is differences in diet across age groups. It has been shown that fish consumption, an essential source of dietary vitamin D, is lower among the younger Norwegian population, and the intake of cod liver oil (rich in vitamin D) increases with age [22, 37, 38].

A seasonal variation in vitamin D status was demonstrated (Table 2) as a higher proportion of vitamin D deficiency was found in subjects who attended from October to February compared to those who attended from March to September. This was presumably a result of the seasonal variation in sun-induced vitamin D production. Seasonal variation in vitamin D status in the population in northern Norway has previously been demonstrated [21, 38].

The reason for performing separate analyses for persons who attended the health examination during October-February and those who attended in March-September was that the high number of measurements during sunnier months might result in a skewed proportion of vitamin D sufficiency. Thus, a separate analysis was done to remove the effect of seasonal sun exposure on the exposure variable for vitamin D status and eliminate the cases where vitamin D sufficiency might only have been temporary. The findings indicated a significant trend across vitamin D status groups. Of those who attended from March to September, it can be interpreted that those who display low vitamin D levels in the summer months are likely to have even lower levels during the winter months. Thus, if vitamin D deficiency increases risks for periodontitis, this should be most apparent among those with a chronically low vitamin D status, i.e., those low during summer. However, this interpretation requires confirmation in further studies with longitudinal designs.

We found that the odds of having at least one periodontal pocket of ≥ 5mm was 78% higher for persons with vitamin D deficiency compared with participants who were vitamin D sufficient even when adjusting for age and smoking, which are known to be some of the most critical factors associated with periodontal pocket depths [9, 10]. This could indicate that periodontal inflammation was increased in those groups where serum 25(OH)D was low or that low vitamin D levels might be a marker of disease activity in this sample—similar to these findings, Alshouibi et al. [39] and Miley et al. [40] found that persons who took vitamin D supplements had fewer and shallower periodontal pockets, although serum 25(OH)D levels were not measured in those studies.

The date of participation was used as a proxy variable for sun-induced vitamin D. Dietary intake data are available in the Tromsø 7 dataset but not included in our analysis. However, the 25(OH)D biomarker is a valid measure of the sum of both diet and sun-induced vitamin D, and it is considered a valid biomarker for vitamin D status [13].

The study has both some strengths and limitations. A strength is the reliability of the collected Tromsø 7 data. The available clinical data on periodontal status and serum 25(OH)D derived from blood samples meant that the possible correlations between individual vitamin D status and indicators of periodontal inflammation could be analysed without relying on self-reported data or estimations. The Tromsø study included many participants from all population groups over 40, with a high participation rate (65%) [30]. Attendees of the dental examination were selected randomly, and very few declined to participate in the dental examination, minimising the risk of selection bias. However, it is not unusual that health study responders are of generally better health than the non-responders, a phenomenon well-examined in previous studies [41–43]. Thus, it is possible that there may be some degree of response bias in the original sample population and that responders of the Tromsø study had better general health and nutrition and higher serum 25(OH)D levels than the non-responders, leading to skewed representation.

A limitation of this study is the cross-sectional study design; neither can causality nor the direction of the relationship between exposure and outcome be determined, even where associations were found. Hence, it is impossible to predict if subjects with low vitamin D levels are more likely to develop more advanced periodontitis or if persons with more advanced periodontitis will be prone to display lower vitamin D levels. One might lead to the other, or both might appear simultaneously for different reasons.

Some of the included covariates were self-reported, meaning there was a risk of both recall and reporting bias, which might have influenced the results of the adjusted analyses. Overall, the contribution of recall and reporting biases in the covariates was small and had a minor effect on the study's internal validity, as the primary variables serum 25(OH)D, periodontitis case classification, and PPD ≥ 5 were assessed and measured by professionals.

## Conclusion

In conclusion, in this Norwegian adult population, blood 25-hydroxy vitamin D [25(OH)D] levels were associated with periodontitis stages II-III/IV and a periodontal pocket depth of ≥ 5 mm in one or more teeth.

Although these findings do not establish a causal relationship between vitamin D deficiency and periodontitis, the associations suggest that vitamin D deficiency might increase periodontitis risk. Further research, using longitudinal or randomised clinical study designs, is needed to confirm these results before recommending maintaining sufficient vitamin D levels as a preventive measure against periodontitis.

## Data Availability

Access to sensitive and personal data, such as the Tromsø Study, is restricted. Upon request, data can be made available to researchers who have previously obtained the approval of the Data- and Publication Committee (DPC) of the Tromsø Study. Information for applicants is available at: https://uit.no/research/tromsostudy.

## Abbreviations

AAP: American Academy of Periodontology
BL: Bone loss
BMI: Body Mass Index
BOP: Bleeding on Probing
cAMP: Cyclic Adenosine Monophosphate
CI: Confidence Interval
D2: Ergocalciferol (Vitamin D2)
D3: Cholecalciferol (Vitamin D3)
EFP: European Federation of Periodontology
HUNT: Helseundersøkelsen i Nord-Trøndelag (The Nord-Trøndelag Health Study)
IBM: International Business Machines
IL-1β: Interleukin-1 beta
IL-6: Interleukin-6
LCMS-MS: Liquid Chromatography-Tandem Mass-Spectrometry
NOK: Norwegian Krone (currency)
NHANES: National Health and Nutrition Examination Survey
OPG: Orthopantomogram
OR: Odds Ratio
PPD: Periodontal Pocket Depth
SACN: Scientific Advisory Committee on Nutrition
SPSS: Statistical Package for the Social Sciences
TNF-α: Tumor Necrosis Factor-alpha
UV: Ultraviolet
VDR: Vitamin D Receptor
25(OH)D: 25-Hydroxyvitamin D
